# Retrospective radiological analysis to establish anatomical variation in angulation between medial canthus and the middle turbinate, for optimal positioning of Lester Jones Lacrimal bypass tubes in treatment of Epiphora

**DOI:** 10.1371/journal.pone.0288856

**Published:** 2023-12-29

**Authors:** Yahya Khedr, Egle Rostron, Colin Vize

**Affiliations:** 1 Hull University Teaching Hospitals and University of Edinburgh, Hull, United Kingdom; 2 Hull University Teaching Hospitals, Hull, United Kingdom; Federal Medical Centre, Asaba, NIGERIA

## Abstract

**Purpose:**

To determine the optimum angle for placement of Lester Jones lacrimal bypass tube using fixed radiological markers on CT scan head with axial and coronal cuts, as well as analysing the anatomical variation and range of angulation between individuals within our local population.

**Methods:**

A retrospective radiological study conducted on a randomly selected sample of 384 adult patients in a UK Teaching Hospital. The angle between the medial canthus and the middle turbinate was measured on CT scans of the head using fixed radiological anatomical landmarks and analysed using the IMPAX software. Patients with orbital or nasal fractures, as well as those with history of surgical procedures involving the facial bones, were excluded. The accuracy of our measurements was validated using three dimensional (3D) CT head reconstruction technology.

**Results:**

Analysis of the results showed a range of angulation between 28–45 degrees, with a mean angle of 36.99 ± 4.78 SD. There was no significant correlation found when comparing the different age groups using the One Way ANOVA test. Furthermore, a non-significant correlation was found between males and females when their mean angles were compared using the independent t-test.

**Conclusion:**

Our study showed that the ideal angle for insertion of Lester Jones tube would be between 30–45 degrees, with a mean of 37 degrees. No significant correlation was found between the age of the patient and the ideal angle of insertion of Lester Jones tube. Moreover, no significant difference was found in the angle measurements between males and females.

## Introduction

Lester Jones (LJ) tube, originally described by Lester T. Jones in 1965, is a lacrimal bypass drainage system used as an alternative pathway for tears from the conjunctival sac to the nasal cavity where the presence of extensive canalicular disease precludes successful outcome from Dacryocystorhynostomy (DCR) alone. The surgical procedure of implanting the LJ lacrimal bypass tube through the conjunctival sac to the nasal cavity, termed Conjunctivodacryocystorhynostomy (CDCR) was traditionally performed together or following a DCR. Dr. Jones describes placement of a guide needle, the point of which should be in the lacus lacrimalis exactly 2mm posterior to the cutaneous margin of the canthal angle, and then pushed to emerge directly posterior to the anterior tear sac flap, and slightly below the level of the palpebral fissure [1]. Although Dr. Jones has not provided recommended angular measurements for his technique, his original paper contains careful illustrations, and the angle measured from the horizontal meridian was found to be approximately 30 degrees [1]. Several years later, Goldberg modified the procedure using a more fornix-based position of the tube which is placed more vertically, allowing better drainage due to the effect of gravitational forces and capillarity [2]. The angle measured from the horizontal line based on illustrations was 45 degrees in this instance.

CDCR without the skin incision, or single puncture technique also termed Conjunctivorhynostomy, has been described subsequently in which the trocar is passed directly via the caruncle through the thin lacrimal bone into the nasal cavity in the absence of a DCR [3]. This approach has an advantage of being faster, avoids external scarring, and some argue that reduced movement of the LJ tube snuggled tightly within the lacrimal bone, results in less risk of tube migration.

LJ tube has a good success rate in treating epiphora, however 43% of cases were reported as needing to be replaced [4]. Extrusion and migration are commonest complications, which can be significantly reduced by the use of Stop-Loss tubes containing a silicone phalange. Moreover, malpositioned LJ tubes can lead to complications which include conjunctival overgrowth, medial or lateral migration, episcleritis, scleral erosion and ulceration, as well as, discomfort and infection [3,5]. A study conducted by Ibrahim HA et al, (2018) regarding single puncture technique for LJ tube insertion discussed the need for re-intervention following the first operation and this is due to several factors which included inadequate site for tube insertion [6].

The empirical recommendation for ideal placement of LJ tube is angulation of 30–45 degrees from the horizontal, however no published data is available on anatomical studies validating this number, or indeed the extent of anatomical variation between individuals in a population. Moreover, no study was found to provide information regarding the influence of age, gender or ethnicity on the surgical success rate of inserting LJ tubes. Our aim was to measure the angle between the medial canthus and middle turbinate as an indicator for optimal positioning of LJ tube using fixed radiological markers, and analyse anatomical variation and a range of angulation between individuals within our local population.

## Methods

The design of the study is a retrospective collection and analysis of empirical radiological data of anatomical landmarks. Studied population was selected as a random sample of adult population from the city of Kingston upon Hull, UK, who had CT scan of the head and sinuses performed with axial sections and coronal reconstruction using thin 1mm cuts. All data was accessed and collected for analysis during the first 6 months of 2020 from IMPAX software. Patients with orbital or nasal fractures, or those who previously underwent surgical procedures involving facial bones, have all been excluded. We have included in our study patients 18 years of age and above. Those under 18 years of age have been automatically excluded.

The required sample size was calculated using Qualtrics statistical analysis tool to ensure that a confidence level of 95% and a margin of error ± 5% is achieved as deemed to be appropriate representation of Hull’s population of 320,825 [7]. Qualtrics statistical calculator has estimated a sample size of 384 patients as adequate to fulfil the above criteria [8].

### IMPAX software system [9] for radiological image analysis has been used to analyse images and for the calculation of angles as follows

**Fig 1:**
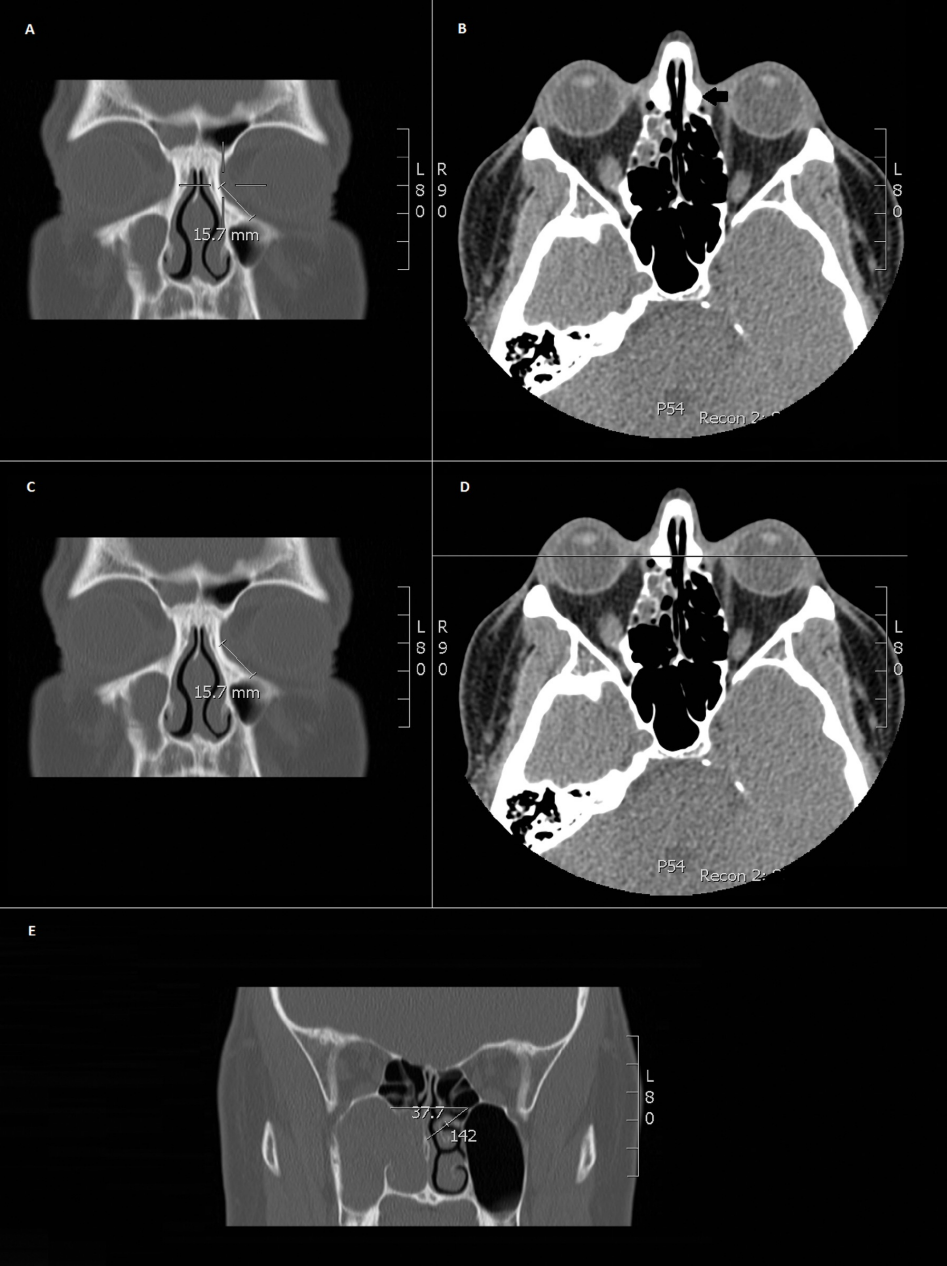
Shows step by step representation of the methodology used to measure the angle. (**A**) CT scan of the head on coronal cut and (**B**) axial cut. The black arrow seen pointing at the anterior lacrimal crest on the axial scan with the linked marker seen on coronal cut. (**C**) and (**D**) show a diagonal line drawn on the coronal cut, joining the beginning of the anterior lacrimal crest (confirmed by the linked marker on the axial cut) to the end of its curve. (**E**) Coronal cut of the head showing the horizontal line joining the midpoints of the diagonal lines on either side, with another line going to the most anterior portion of the axilla of the middle turbinate, and the angle present between them (Khedr, Y).

1. Anterior lacrimal crest was identified on the axial CT scan of the head and a radiological marker was placed, which was subsequently linked, in order for the marker to appear on the coronal reconstruction section (**Fig 1A and 1B**).
2. A diagonal line has been drawn along the curvature of the anterior lacrimal crest, which has aided identification of its midpoint as visualized on the coronal section (**Fig 1C and 1D**).
3. A horizontal line has been drawn to join the midpoint of the anterior lacrimal crest curvature to its counterpart on the contralateral side on an axial cut showing the most anterior portion of the axilla of the middle turbinate (**Fig 1E**).
4. Using the angle measurement tool, which is available within the IMPAX software system, an additional line has been drawn from the midpoint to the most anterior portion of the axilla of the middle turbinate, which has aided the identification of the required angle as illustrated (**Fig 1E**).
5. In order to verify that the methods in which we collected our data was accurate and reproducible, we measured the angle between the medial canthus and the middle turbinate in a random patient using the same method mentioned in this study but on a 3D reconstruction of the CT Scan head and comparing it to the coronal section of the same patient.

### Outcome measures

- Finding the anatomical angle at which the LJ tube can be inserted with the least possibility of complications.

### Primary outcome

- Measuring the angle between the medial canthus and middle turbinate as a surrogate marker for optimal positioning of a LJ tube.

### Secondary outcome

- Analysing anatomical variation and the range of angulation between individuals within our local population.

### Statistical analysis

- Calculating the mean, median and mode for the angle between the medial canthus and the middle turbinate in the selected sample.
- Comparing the mean angle in different age groups in the selected sample.
- Comparing the mean angle between males and females.

### Ethical statement

The data within the study have been pseudoanonymised to comply with local clinical governance regulations and ethics committee, thus informed consent was waivered due to the absence of patient identifiable details.

## Results

The study included 384 radiological images of adult patients who had CT scans of the head using both axial and coronal cuts (153 males and 231 females). The age of the patients ranged from 18–82 years old with a mean of 46.72 ± 14.05 SD as seen in the table in **Table 1**. The range of angulation between the medial canthus and middle turbinate detected in this sample was 28–45 degrees with a mean of 36.99 ± 4.78 SD. The median was calculated to be 36.65 degrees and the mode as being 45 degrees.

**Table 1:** Shows the distribution of patients according to their gender, age and analysis of the angle detected between the medial canthus and middle turbinate.

		No. = 384
Gender	Males Females	153 (39.8%) 231 (60.2%)
Age	Mean ± SD	46.72 ± 14.05
	Range	18–82
Angle	Mean ± SD	36.99 ± 4.78
	Mode	45
	Median (IQR)	36.65 (32.55–41.0)
	Range	28–45

**Fig 2:**
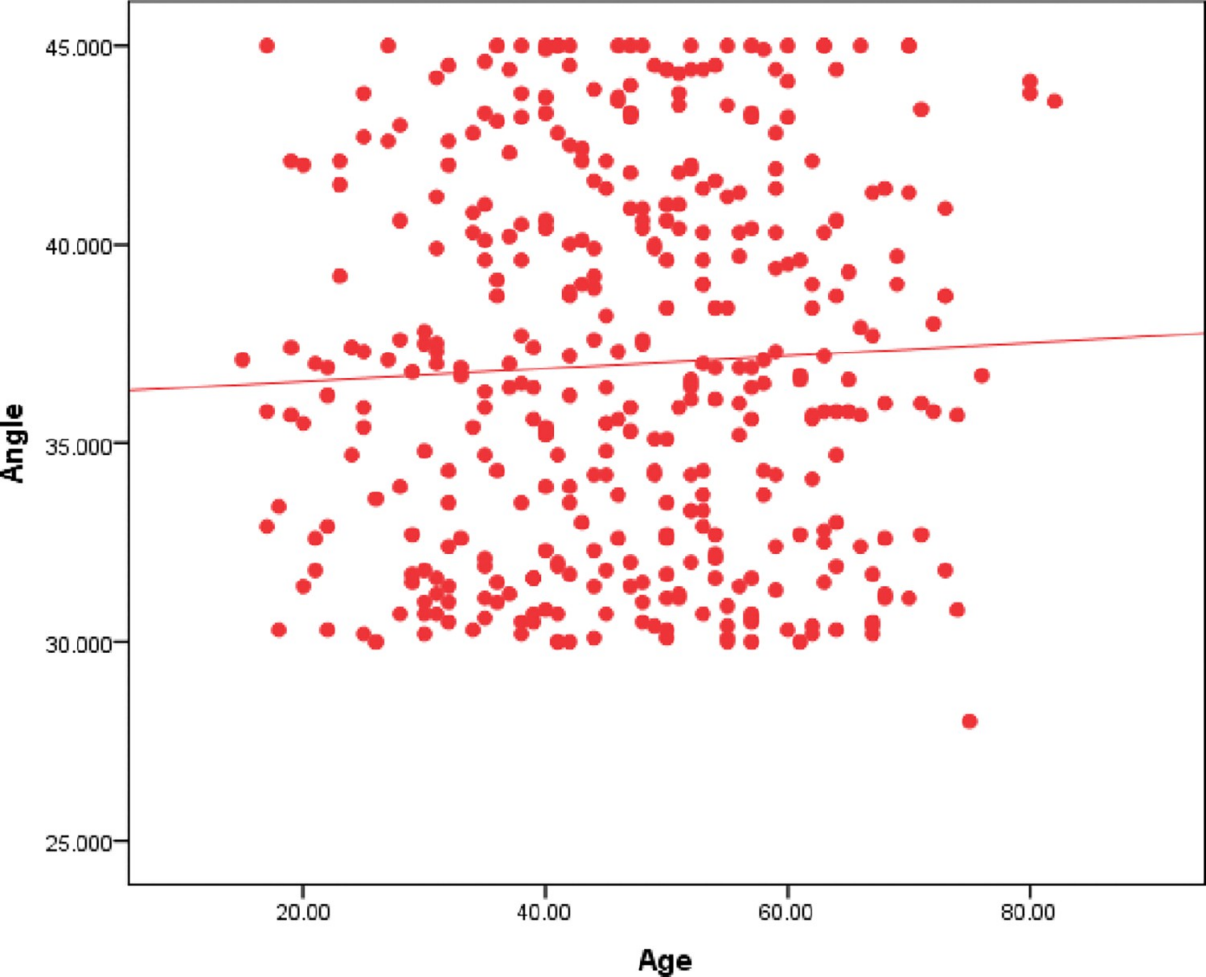
Shows a scatter plot graphical representation of all the patients’ angles against the age of the patients.

The angle between the medial canthus and the middle turbinate of all the patients was plotted on a scatter plot graph against the age of the patients which showed the distribution of the angles in a range between 30–45 degrees with one outlier of 28 degrees as seen in **Fig 2**.

**Fig 3:**
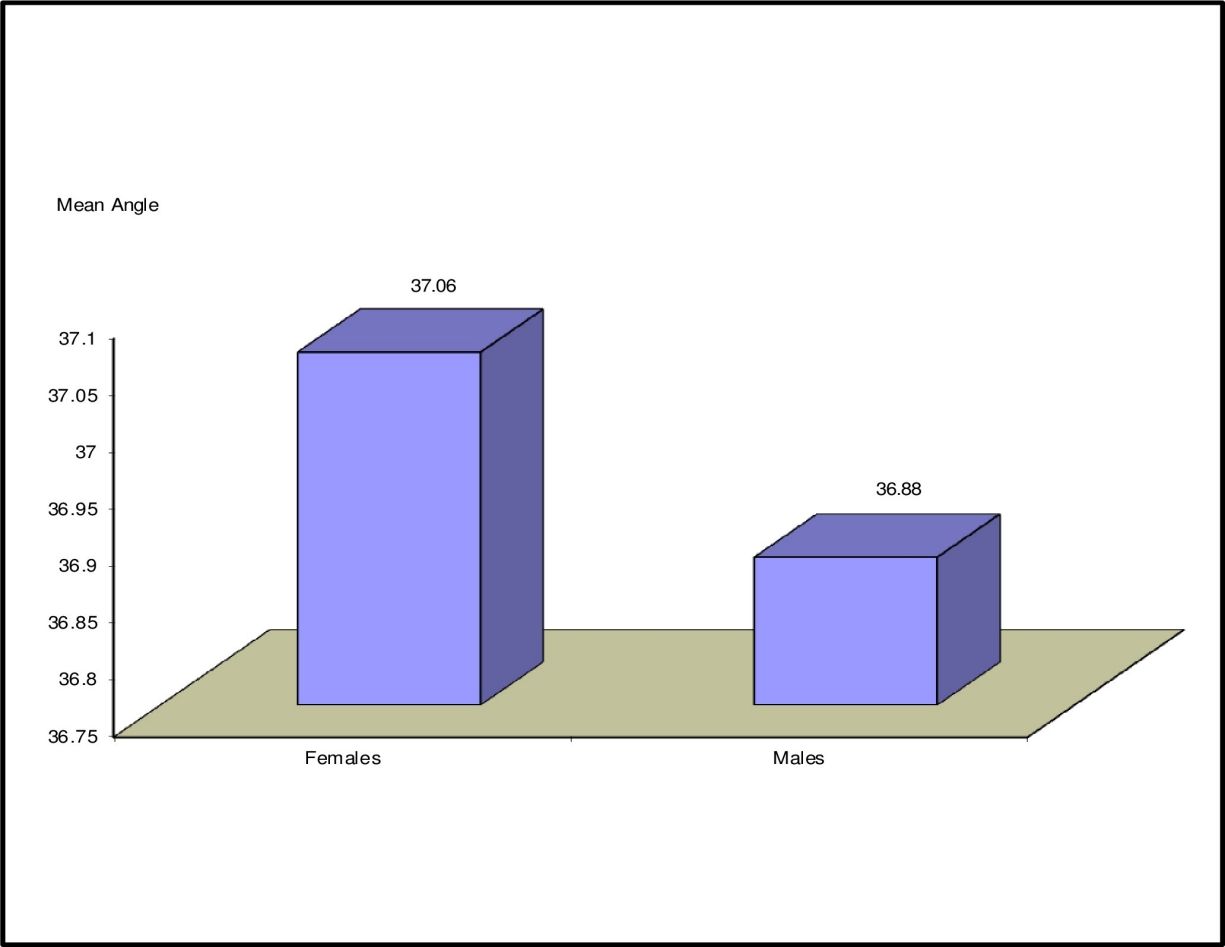
Shows a graphical representation of the mean angle in both males and females groups.

The percentage of males and females in the study is 39.8% and 60.2% respectively, with a male to female ratio of 2:3. The mean, median and range of angles for both the males and females groups were calculated. The female group had a mean of 37.06 ± 4.62 SD, while the male group had a mean of 36.88 ± 5.02 SD as shown in **Table 2**, with a difference of 0.18 degrees between both groups. With regards to the median, it was found to be 36.7 degrees in the female group while it was 36.5 degrees in the male group, with a difference of 0.2 degrees. The range of angles in females was 30–45 degrees and males was 28–45 degrees. Independent t-test was used to compare both groups, with a p-value of 0.728 showing a non-significant correlation between them as shown in **Fig 3**.

**Table 2:** Shows the mean, median and range angles for both males and females groups and comparing the results using Independent t-test to show the significance.

Angle	Females	Males	Test value (Independent t-test)	P-value	Significance
	No. = 231	No. = 153			
Mean ± SD	37.06 ± 4.62	36.88 ± 5.02	0.382	0.728	NS
Median (IQR)	36.7 (32.9–40.6)	36.5 (31.8–41.8)			
Range	30–45	28–45			

**Fig 4:**
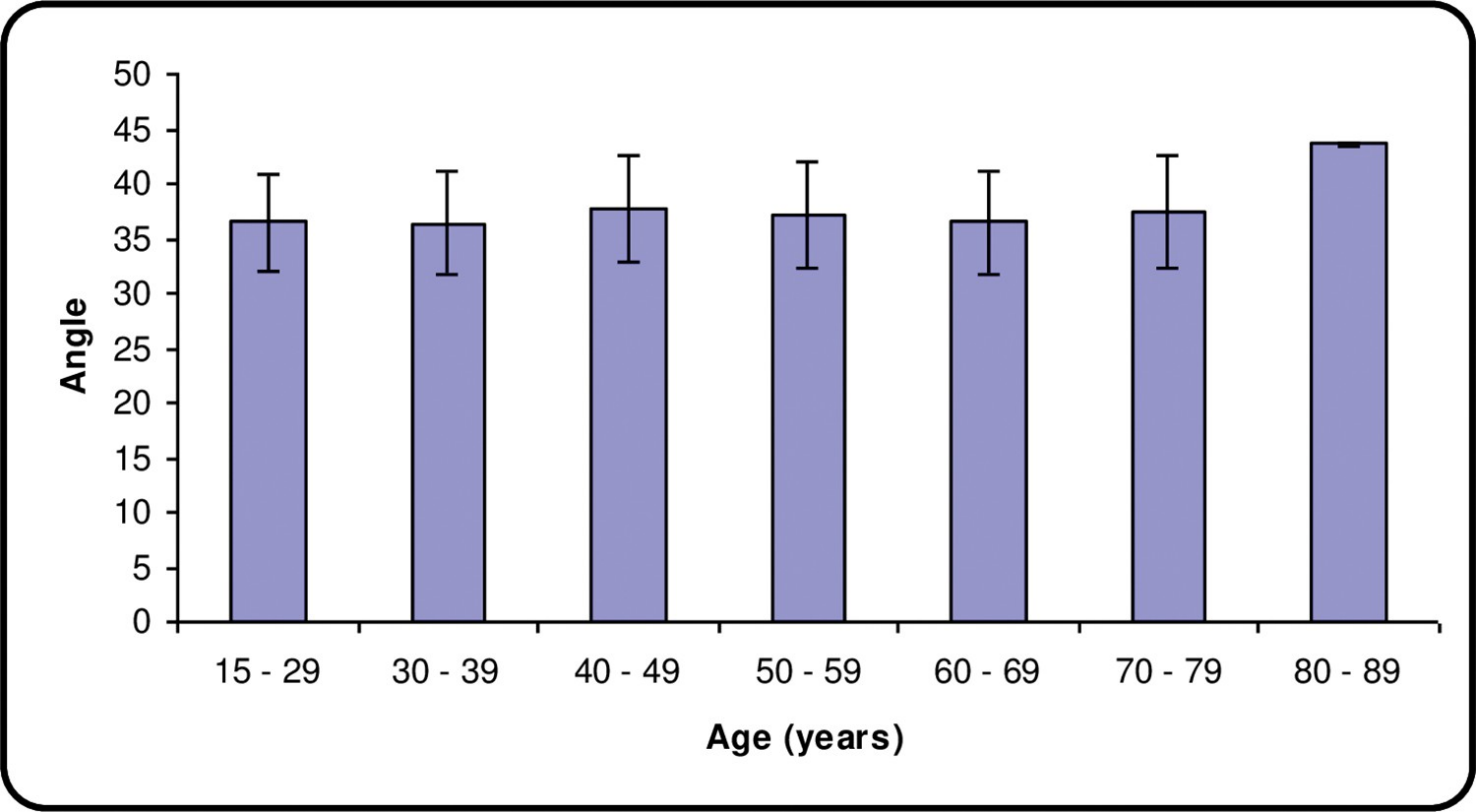
Shows a graphical representation of the mean angle ± standard deviation for each age group.

The mean angles were similar in age groups 18–29, 30–39 & 60–69, with an increasing angle in ascending order in the age groups 50–59, 70–79 & 40–49. However, the age group 80–89 showed the highest mean detected at 43.70 ± 0.14 SD as seen in **Table 3**. The range of angles was the same in all age groups at 30–45 degrees except for 2 age groups; the 70–79 age group showing a range of 28–45 degrees and the 80–89 age group which showed a ranged of 43.6–43.8 degrees. These results were compared using the One Way ANOVA test and a P-value was calculated at 0.215 which showed a non-significant correlation as shown in **Fig 4**.

**Table 3:** Shows the stratification of patients into age groups with the mean and range calculated for each age group and the results compared using One Way ANOVA test.

Age groups	Mean ± SD	Range	Test value (One Way ANOVA)	P-value	Significance
18–29	36.46 ± 4.37	30–45	1.395	0.215	NS
30–39	36.41 ± 4.74	30.2–45			
40–49	37.66 ± 4.91	30–45			
50–59	37.13 ± 4.80	30–45			
60–69	36.45 ± 4.68	30–45			
70–79	37.43 ± 5.20	28–45			
80–89	43.70 ± 0.14	43.6–43.8			

**Fig 5:**
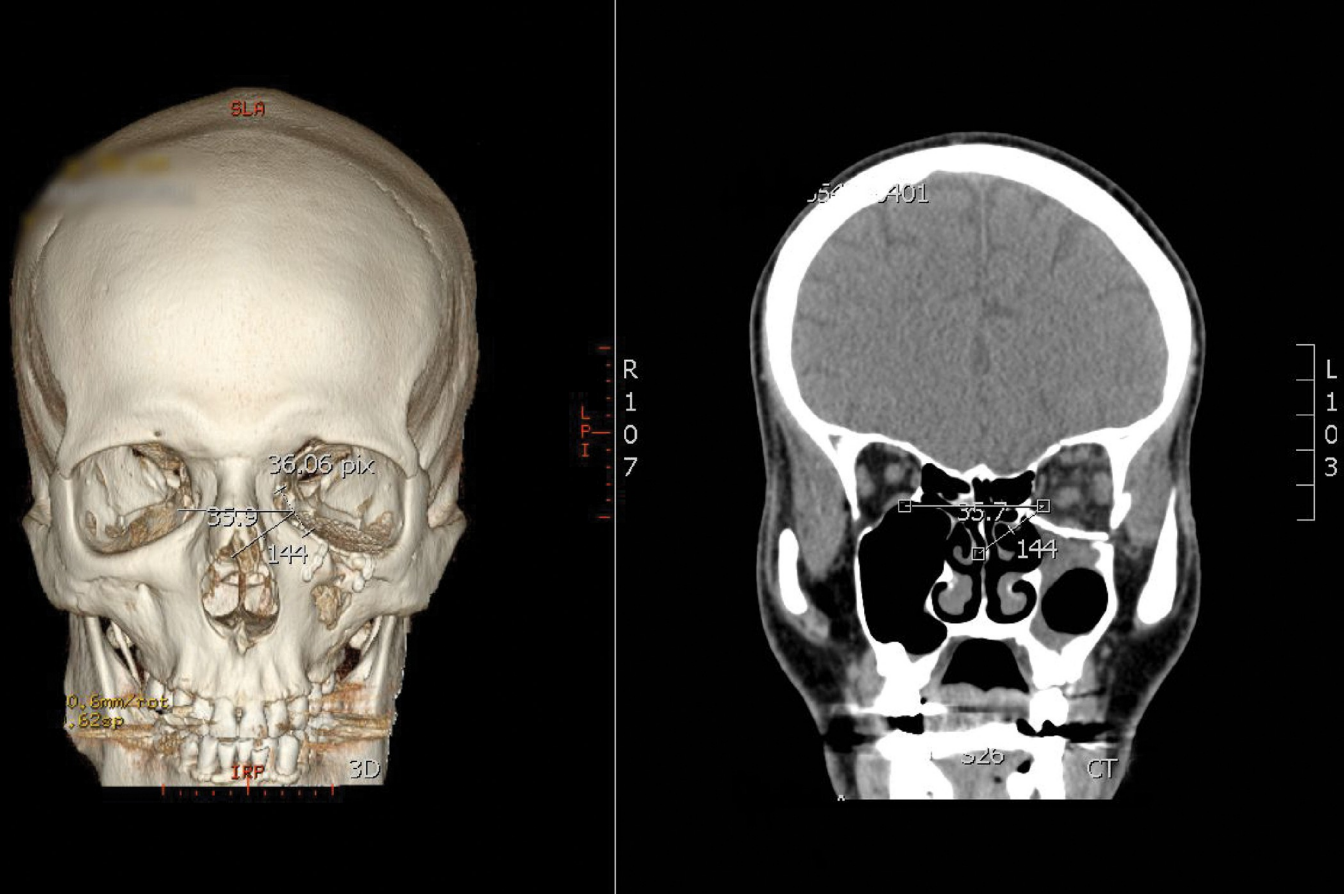
Shows a 3D reconstruction of a CT scan head in anteroposterior view (left) with the angle between the medial canthus and the middle turbinate measured on it and a coronal cut of the same CT scan head (right) with the angle measured on it (Khedr, Y).

The method was standardized in both the 2D and the 3D representations of the CT Head and provided us with an almost identical angle measurements at 35.7 degrees in the 2D coronal cut, in comparison to the 3D reconstruction which showed an angle of 35.9 degrees as seen in **Fig 5**, showing an 0.2 degrees difference between both methods thus proving the accuracy of our methods of measurement of the angle.

## Discussion

In this radiological study, we aimed at addressing the gap in current published literature regarding the evidence to support the recommended angle of insertion of LJ tube from the anatomical standpoint.

A study by Rose & Welham as a 25 years’ experience in the use of Lester Jones tube, recommended that the ideal placement of LJ tube is angulation of 30–45 degrees, as a more horizontally placed tube would lead to a higher rate of complications secondary to inappropriate tube position and a greater risk of extrusion [5]. Our results compare favourably with these findings. Moreover, from physics point of view, capillarity is aided with gravitational forces in vertically placed tubes rather than horizontal placed ones where gravitational forces are theoretically eliminated [10].

We looked specifically at age and gender differences between our studied subjects. This interest was based on previous studies that have analysed anatomical changes in the orbital structures between males and females, as well as the effect of ageing [11–13]. We found no statistically significant difference between the gender groups in our study. The ratio of females to males was 3:2 with females at 60% and males at 40%, however, this percentage of female predominance was unbiased as the sample selection was by simple random sampling from our database of adult patients who had CT scan head with axial and coronal cuts. Our data has shown that there was no correlation in the mean angle in both males and females; with a difference of 0.18 degrees between both groups, which was statistically non-significant.

When comparing the mean angle according to age groups, we have stratified the sample into a 10 years age groups with addition of the 18 & 19 years old to the first age group due to their low numbers. It was concluded that there was no significant correlation when comparing the different age groups in the study using the one way ANOVA test.

A study on the nasal cavity and paranasal sinus bony variations using computed tomography which was conducted on 172 adult patients, concluded that there was no clinically significant correlation between the age of the patients and the anatomical variation, as well as no statistically significant difference between both genders [14].

Whilst our study does not replace the need to individualise the placement of LJ tube by direct visualisation of the distal exit point using an endoscope, it does substantiate and confirm that the current practice is in keeping with anatomical findings. Furthermore, it assists the surgeon in adopting the appropriate initial angulation at the medial canthus in order to have an increased chance of exiting into the nasal cavity in the region of the middle turbinate. From our study it is apparent that anatomical variation between different genders and age groups does not appear to make a significant difference.

Radiological evaluation of anatomical markers can be useful in studying oculoplastic, orbital and lacrimal anatomy, particularly the bony structures as these show up best on a CT scan. A study conducted by Schiff *et al*. [15] has used computed tomography to assess volume change after endoscopic orbital decompression for Grave’s Ophthalmopathy, and another by Lock *et al*. [16] used CT scan analysis to assess anterior maxillary wall and lacrimal duct relationship in Orientals for prelacrimal access to the maxillary sinus.

In our study we have used two dimensional CT images in which the views were inter-changed between axial cuts and coronal reconstruction, in order to aid identification of specific anatomical landmarks. The method used to measure the angle using the IMPAX software for radiological analysis was formulated after several attempts of trial and error to produce the ideal method for this measurement, which can be repeatable and comparable using bony landmarks. Since the medial canthus can not be anatomically localised on a CT scan, we have used the midpoint of the anterior lacrimal crest as our radiological landmark to represent the medical canthus. The middle turbinate was used as our second radiological landmark due to the fact that the LJ tube should be resting anterior to it, for optimal positioning [1,2]. In order to avoid any element of error and to provide consistency to the results, all the measurements were performed by the same author (Khedr, Y).

Limitations of our study include its retrospective nature and use of angulation between medial canthus and middle turbinate as a surrogate marker of ideal LJ tube angulation, in contrast to a potential prospective study in which a radiological measurement of the angle for patients listed for CDCR were to be performed preoperatively and postoperatively with the Lester Jones tube in situ. This is however a basis for future studies.

It could be argued that 3D CT reconstructions of all the images studied would have been the gold standard of radiological analysis, but such expert technique is time and expertise dependant, and would be considered very costly within a busy NHS hospital.

Lack of ethnic background information may be considered another limitation, which has arisen due to the pseudoanonymised nature of our data. Furthermore, the majority of Hull’s population are of a Caucasian background [7] and thus even if we had the required information about the ethnicity, there would have still been some degree of bias in the data.

We also came across another limitation while conducting this study which was related to the randomisation of our sample selection of 384 patients. During the planning phase of our study it was decided that the most suitable mean of randomisation was simple randomisation which was done by simple random selection of patients from our data base regardless of age or gender, after making sure they did not have any of our exclusion criteria. According to a study performed as an overview on the randomisation techniques in clinical research and concluded that simple randomisation techniques is problematic in studies with a small sample size [17], however, in studies like ours with a larger sample size this should not affect the results nor make them biased towards one particular group.

Finally, this is not necessarily a limitation but a point of interest that could be improved in future studies, where our measurement of the angle was done manually using the set anatomical landmarks on the CT scans with the aid of the angle measurement tool in the IMPAX software. Even though it was all measured by the same person using the same method in all the patients, which decreases the risk of bias and error, however, some people may argue that this could be a setback in the measurements as it puts in perspective the factor of human error.

In conclusion:

- Our data showed that the ideal angle for insertion of LJ tube would be between **30–45 degrees**, with a **mean of 37 degrees**, which confirmed the predetermined hypothesis.
- No significant correlation was found between the age of the patient and the ideal angle of insertion of LJ tube.
- No significant difference was found in the angle measurements between males and females.

It is our recommendation that the ideal angle for insertion of LJ tube would be roughly 37 degrees but bearing in mind that it can lie anywhere between 30–45 degrees depending on the anatomical variations and thus nasal endoscopy is a crucial step in confirming that the nasal end of the tube is in the correct positioning.

## References

[1] Jones LT. Conjunctivodacryocystorhinostomy. American Journal of Ophthalmology. 1965;59(5):773–83. doi: 10.1016/0002-9394(65)93004-7 14288913

[2] Schwarcz RM, Lee S, Goldberg RA, Simon GJ. Modified conjunctivodacryocystorhinostomy for upper lacrimal system obstruction. Archives of Facial Plastic Surgery. 2007;9(2):96–100. doi: 10.1001/archfaci.9.2.96 17372062

[3] Devoto MH, Bernardini FP, de Conciliis C. Minimally invasive conjunctivodacryocystorhinostomy with Jones tube. Ophthalmic Plastic and Reconstructive Surgery. 2006;22(4):253–5. doi: 10.1097/01.iop.0000226861.02781.af 16855494

[4] Bagdonaite L, Pearson AR. Twelve-Year Experience of Lester Jones Tubes—Results and Comparison of 3 Different Tube Types. Ophthalmic plastic and reconstructive surgery. 2015;31(5):352–6. 25369837 10.1097/IOP.0000000000000340

[5] Rose GE, Welham RAN. Jones’ lacrimal canalicular bypass tubes: Twenty-five years’ experience. Eye. 1991;5(1):13–9. doi: 10.1038/eye.1991.3 2060661

[6] Ibrahim HA, Sabry HN, Ibrahim AA. The single-puncture technique for guided Lester Jones tube insertion. Journal of the Egyptian Ophthalmological Society. 2014;107(4), 258–262.

[7] WPR. Hull Population 2020 (Demographics, Maps, Graphs) 2020 [Available from: https://worldpopulationreview.com/world-cities/hull-population/.

[8] Qualtrics. Sample Size Calculator 2019 [Online]. Qualtrics. Available from: https://www.qualtrics.com/blog/calculating-sample-size/ [Accessed 23/02/2020].

[9] IMPAX. IMPAX 6: Because all PACS are not created equal 2007 [Available from: http://www.agfahealthcare.com/he/germany/de/binaries/IMPAX_6_tcm602-90706.pdf.

[10] Bitten JF, Fochtman EG. Flow of liquids in horizontal capillary tubes. American Institute of Chemical Engineers Journal. 1963;9(2):279–82.

[11] Weaver AA, Loftis KL, Tan JC, Duma SM, Stitzel JD. CT Based Three-Dimensional Measurement of Orbit and Eye Anthropometry. Investigative Ophthalmology & Visual Science. 2010;51(10):4892–7. doi: 10.1167/iovs.10-5503 20463322

[12] Kaye SB, Green JR, Luck J, Lowe KJ. Dependence of ocular protrusion, asymmetry of protrusion and lateral interobital width on age. Acta ophthalmologica. 1992;70(6):762–5. doi: 10.1111/j.1755-3768.1992.tb04884.x 1488885

[13] Kashkouli MB, Nojomi M, Parvaresh MM, Sanjari MS, Modarres M, Noorani MM. Normal values of hertel exophthalmometry in children, teenagers, and adults from Tehran, Iran. Optometry and Vision Science. 2008;85(10):1012–7. doi: 10.1097/OPX.0b013e3181890dc7 18832980

[14] Kayalioglu G, Oyar O, Govsa F. Nasal Cavity and Paranasal Sinus Bony Variations: A Computed Tomographic Study. Rhinology. 2000;38(3):108–13. 11072655

[15] Schiff BA, McMullen CP, Farinhas J, Jackman AH, Hagiwara M, McKellop J, et al. Use of Computed Tomography to Assess Volume Change After Endoscopic Orbital Decompression for Graves’ Ophthalmopathy. American Journal of Otolaryngology. 2015;36(6):729–35. doi: 10.1016/j.amjoto.2015.06.005 26545461

[16] Lock PSX, Siow GW, Karandikar A, Goh JPN, Siow JK. Anterior Maxillary Wall and Lacrimal Duct Relationship in Orientals: CT Analysis for Prelacrimal Access to the Maxillary Sinus. European archives of Otorhinolaryngology. 2019;276(8):2237–41. doi: 10.1007/s00405-019-05446-0 31049653

[17] Lachin JM, Matts JP, Wei LJ. Randomization in clinical trials: conclusions and recommendations. Control Clinical Trials. 1988;9(4):365–74. doi: 10.1016/0197-2456(88)90049-9 3203526

